# Different adaptive strategies in *E. coli* populations evolving under macronutrient limitation and metal ion limitation

**DOI:** 10.1186/s12862-018-1191-4

**Published:** 2018-05-18

**Authors:** Omar M. Warsi, Dan I. Andersson, Daniel E. Dykhuizen

**Affiliations:** 1 Department of Ecology and Evolution, Stony Brook University, 650 Life Sciences Building, Stony Brook, NY 11794 USA; 2 Department of Medical Biochemistry and Microbiology, Uppsala University, Uppsala, Sweden

**Keywords:** Nitrogen limitation, Magnesium limitation, Experimental evolution, Low nutrient environment

## Abstract

**Background:**

Adaptive responses to nutrient limitation involve mutations that increase the efficiency of usage or uptake of the limiting nutrient. However, starvation of different nutrients has contrasting effects on physiology, resulting in different evolutionary responses. Most studies performed to understand these evolutionary responses have focused only on macronutrient limitation. Hence our understanding of adaptation under limitation of other forms of nutrients is limited. In this study, we compared the evolutionary response in populations evolving under growth-limiting conditions for a macronutrient and a major cation.

**Results:**

We evolved eight populations of *E. coli* in nutrient-limited chemostats for 400 generations to identify the genetic basis of the mechanisms involved in efficient usage of two nutrients: nitrogen and magnesium. Our population genomic sequencing work, based on this study and previous work, allowed us to identify targets of selection under these nutrient limiting conditions. Global transcriptional regulators *glnGL* were targets of selection under nitrogen starvation, while proteins involved in outer-membrane biogenesis (genes from the *lpt* operon) were targets of selection under magnesium starvation. The protein involved in cell-cycle arrest (*yhaV*) was a target of selection in both environments. We re-constructed specific mutants to analyze the effect of individual mutations on fitness in nutrient limiting conditions in chemostats and in batch cultures. We further demonstrated that adaptation to nitrogen starvation proceeds via a nutrient specific mechanism, while that to magnesium starvation involves a more general mechanism.

**Conclusions:**

Our results show two different forms of adaptive strategies under limitation of nutrients that effect cellular physiology in different ways. Adaptation to nitrogen starvation proceeds by upregulation of transcriptional regulator *glnG* and subsequently of transporter protein *amtB*, both of which results in increased nitrogen scavenging ability of the cell. On the other hand, adaptation to magnesium starvation proceeds via the restructuring of the cell outer-membrane, allowing magnesium to be redistributed to other biological processes. Also, adaptation to the chemostat environment involves selection for loss of function mutations in genes that under nutrient-limiting conditions interfere with continuous growth.

**Electronic supplementary material:**

The online version of this article (10.1186/s12862-018-1191-4) contains supplementary material, which is available to authorized users.

## Background

Nutrient starvation is an important selective pressure experienced by many organisms in different ecosystems [1–5] and organisms adapt to limitations of these nutrients through different strategies. Many studies have explored the ecological and evolutionary characteristics of these strategies, shedding light on topics of species co-existence, community dynamics, rates of adaptation and population equilibrium points [6–12]. Physiologically, these nutrients fall into three categories: macronutrients, major cations and micronutrients [13]. Macronutrients form the backbone of all biomolecules essential to the cell; hence their universal importance is obvious and well understood. Major cations and micronutrients, on the other hand, play important roles as cofactors for enzymes, osmotic solutes and as stabilizing cations for biomolecules. Among these it is assumed that only the metal ions of zinc and magnesium are universally vital, with the essentiality of other metals being more species-specific [13]. Given the different roles these nutrients play in the cell, it is no surprise that their limitation results in very different selective pressures for the organism.

Although the importance of these different classes of nutrients to cellular physiology has long been appreciated [14], only recently has the genetic basis underlying the adaptive strategies for their efficient usage been elucidated. Most work in this area employs an experimental evolution approach, with a strong focus on macronutrients like carbon limitation [15–17], nitrogen limitation [18, 19] or phosphorous limitation [20]. A common theme occurring in these macronutrient limitation studies is selection for mutations that result in an increased uptake of the limiting nutrient. On the other hand, studies investigating adaptation under starvation of other two classes of nutrients i.e. major cations and micronutrients are very limited. To the best of our knowledge only two studies have used experimental evolution to study the general evolutionary response to limitation of metal ions (cobalt metal ion limitation by Chou et al. [21]; Fe limitation by Walworth et al. [22]). In the first of these studies, the evolutionary response to cobalt limitation for populations of *Methylobacterium extorquens* was shown to select for increased uptake of cobalt caused by increased expression of transporter proteins. The second study looked at long-term adaptation occurring under co-limitation of Fe/P in cyanobacteria *Trichodesmium* as compared to single nutrient limitations of Fe or P. Proteomic analysis revealed an enrichment of Iron stress-response genes in both the single nutrient limiting Fe and Fe/P co-limiting environments; the genetic basis underlying the enrichment was not determined. Besides these two studies, there is a significant body of literature where evolutionary dynamics under limitation of iron has been investigated in microbial populations that release iron-scavenging proteins. These studies have focused on interaction between different microbial populations and on the evolutionary response that is generally mediated through these iron-scavenging proteins [23, 24]. Thus, even though transporter and scavenging proteins were shown to be important targets of selection in these studies, given their limited number, our understanding regarding the genetics of adaptive strategies under limitation of metal ions is still sketchy.

Consequently, our work was designed to understand and contrast the evolutionary response in *E. coli* populations selected under the different nutrient limiting regimes of a macronutrient and a metal ion, specifically, nitrogen and magnesium. Nitrogen is central macronutrient that is essential for formation of most biomolecules. Nitrogen limitation results in the induction of the *glnGL (*NtrC-NtrB) regulon in most prokaryotes, which induces large numbers of nitrogen-scavenging proteins [25, 26] and increased transcription of catabolic genes like *ast* (arginine degrative enzymes) and *gab* (GABA degradative enzymes) [25]. Previous work on nitrogen starvation has shown transporter proteins to be important targets of selection [18, 19]. On the other hand, magnesium is an essential major cation that is a vital cofactor for multiple enzymes and for ribosomes as well as plays an important role in stabilizing the outer cell membrane. Magnesium limitation induces the PhoPQ regulon leading to phenotypic changes like biofilm formation, reduced cell motility in bacteria and altered turnover rate of ribosomes [27]. Magnesium starvation is also experienced by pathogenic cells in macrophages that cause physiological changes resulting in reduced susceptibility to the host immune system and to antibiotics [27].

Our experimental design consisted of eight independent *E. coli* populations evolved under different nutrient limiting conditions in chemostats for a period of 400 generations (four under each nutrient limitation). We previously showed that over the course of our experiment these populations had an increased fitness compared to the ancestral strain [28]. Population sequencing of these evolved populations at generation 400 allowed us to identify the important targets of selection under these contrasting nutrient limiting conditions; mutations in transcriptional regulator *glnGL* was selected under nitrogen limiting conditions while mutations in genes *yhaV*, *phoQ* and *lptG* were selected under magnesium limiting conditions [28]. In this study, to further our understanding of adaptive evolution under these nutrient-limiting conditions, we sequenced all eight evolved populations from the mid-point of our experiment (generation 168). Comparison of adaptive mutations over the two time-points identified which target genes and mutations could increase fitness under these nutrient-limiting conditions. By reconstructing strains with individual mutations, our results showed that there are at least two different adaptive strategies involved in adaptation to nutrient limitation. Populations evolving under limiting nitrogen conditions acquired mutations in the genes *glnGL*, a two-component global gene regulatory system that responds to and controls the carbon:nitrogen ratio in the cell; while populations evolving under limiting magnesium conditions acquire mutations in genes involved in cell membrane biogenesis. We also demonstrated how clonal interference affected evolutionary outcomes under these selective pressures, and finally, using qPCR analysis and lipopolysaccharide (LPS) hydrophobicity measurements, the mechanistic basis underlying these adaptive genetic changes was explored.

## Methods

### Strain and media used

The ancestor used in the study is a derivative of *E. coli* K-12 MG1655. It is cured of lambda phage and contains no plasmid. It also contains a deletion in a region of its lactose metabolizing operon making it *lac-* and a mutation in the *rpoS* gene making it *rpoS-*. Previous experimental evolution studies performed under nutrient limiting conditions have found *rpoS* mediated pathways to be targets of selection. Mutations in this gene resulted in complicated evolutionary dynamics and for selection of *rpoS-* mutants very early in the evolution experiments [29]. The use of an *rpoS-* mutant as an ancestor for all the evolution experiments in this study was done to get around these complicated dynamics and to importantly disentangle the general evolutionary response mediated via *rpoS* from the more specific nutrient starvation responses.

Glucose minimal media M9 with different concentrations of salts was used for the long-term evolution experiments. In general, minimal M9 media was made by adding 1.75 g potassium dibasic phosphate, 0.5 g potassium monobasic phosphate, 1 g ammonium sulphate, 0.5 g sodium citrate and 0.1 g magnesium sulphate in 1 l of water. Water used for the experiments was passed through an de-ionizer and was then distilled. The sugar used in all the experiments was glucose at a concentration of 1 g/L. The concentration of magnesium and nitrogen used in the evolution experiments were based on previously done experiments [28]. Briefly, cells were allowed to grow till stationary phase in flasks at different concentrations of magnesium and ammonium ions. The population density at stationary phase and the indophenol assay for the detection of the ammonium ion was then used to identify concentrations of magnesium and nitrogen that were growth limiting. For nitrogen starvation experiments ammonium ion was used at a concentration of 0.05 g/L (0.7 mM). Sodium sulphate was used to compensate for sulphate concentrations (0.9 g/L). For magnesium ion starvation experiments, no magnesium sulphate was added in the media. Chemostats were changed every 10 days to avoid wall effects. The dilution rate of the chemostat was maintained at 0.33/h, which results in a ~ 2 h generation time. Samples were taken every 24 h and were frozen as glycerol stocks at − 80 °C. Contamination checks were performed every 24 h by plating the samples on citrate plates, with no growth being expected during the course of our experiment. The experiments were allowed to run for 34 days, that equaled ~ 400 generations.

### Fitness assays for evolved clones

To measure relative fitness of evolved clones competition was carried out with the ancestral strain using *lac* operon as the neutral marker (i.e. ability to grow on media plates with lactose as sole carbon source), as has been described before [28]. Briefly, competition experiments were carried out in chemostats under appropriate nutrient conditions using equal proportions of each strain. Selection coefficient was calculated by plotting log of ratios of cell counts to time and calculating the slope of linearly regressed line. Relative fitness was measured as (1 + selection coefficient). Each competition experiment was done in a duplicate. Error bars represent standard errors to the mean. Students-t test was performed to look for statistically significant differences.

### Population whole-genome sequencing

We constructed genomic libraries from DNA extracted from these populations taken at time point of 168 generations, as was done previously for populations taken from the end point of the experiment [28]. Briefly, populations were re-grown using their glycerol stocks in appropriate nutrient limited conditions for 24 h, allowing the population to reach a steady state (constant continuous growth phase) in chemostats. DNA was extracted from 5 ml of this media using the DNeasy blood and tissue kit from Qiagen. Protocols were followed as mentioned in the manual, except for increasing the lysis time to 1 h. Libraries were made using Illumina’s NexteraXT sample preparation kit. Samples were dual-indexed, pooled together and run on Illumina’s Miseq using the Miseq reagent kit v2 (2 × 250 cycle). An average of 75% of reads were above the Q30 score. The coverage for all the samples ranged from 7X to 30X.

### Next-generation sequencing analysis

Geneious was used to map the reads onto the reference genome and to find SNPs. Conservative values, allowing 0.01 probability of a wrong call, were used for trimming the raw reads (40 bp from either end), aligning these reads and for finding variants in the data. The reads were aligned to *E.coli* K-12 MG1655 reference genome, which was downloaded from Genbank (NC_000913.3). The ancestral strain was also sequenced using the same protocol to identify mutations present in the ancestral strain in comparison to the reference genome. These were excluded from the analysis. For mutation detection, the cut-off values used were a minimum coverage of 15× and a minimum mutation frequency of 35%. For comparison of mutations between the two points for each population sample, each mutation was crosschecked manually at the alternate time point i.e. for each gene showing mutations reaching more than 35% in the population at a given time point, the aligned sequence reads at the alternate time point for the same genetic location was searched to see if the given mutation was present. This allowed us not to miss potentially low frequency mutations in our analysis. From this data set, genes were selected as potential targets of selection if these showed mutations i) in at least two replicates under the same nutrient limitation, ii) and were found at both time points in the same population, or iii) reached high frequencies in the population. To confirm the presence these mutations, Sanger sequencing was also performed on isolated clones. breseq (version 0.27.1a) analysis was also performed on the data to detect structural rearrangements or transposon movements [30]. Besides looking for transposon movements in the annotated results, we also looked for signatures of potential movements in the ‘unassigned new junction’ category, which resulted in the identification of mutations in gene *yhaV* under both nitrogen limiting and magnesium limiting conditions.

### Mutant strain construction, growth rate and measurements of selective coefficients

To assess the effect of different mutations on relative fitness, strain construction was performed using a λ-red recombineering technique [31]. The *E.coli* K-12 strain, which had been used for the construction of the KEIO collection, was used as the starting strain for construction of mutants. This allowed us to analyze the growth rate of different deletion mutants from the KEIO collection itself. Briefly, a *cat-sacB-yfp* cassette was PCR amplified from a plasmid using primers that consisted of 45 bp overhangs identical to sequence of the region where the SNP had to be inserted. This PCR product was then electroporated in a strain carrying the λ-red plasmid, and the transformants were selected on chloramphenicol. The 45 bp overhang region allows the PCR product to be recombined into the desired target position. Oligos containing the desired mutation were then electroporated into these transformants and negative selection on sucrose was performed. The desired mutation was confirmed using Sanger sequencing. P1 transduction was used in the final step, where the *cat-sacB-yfp* cassette and the oligo, with the desired mutation, were introduced in a clean background. SNPs were confirmed using Sanger sequencing. Exponential growth rates for these mutants were measured using five biological replicates, each having two technical replicates using a Bioscreen plate reader. The optical density (OD) values obtained were log transformed, and the slope of log OD to time was used as exponential growth rates. Mutants with individual adaptive SNPs were also competed in chemostats with the wild type allele using fluorescent markers. Briefly, yellow fluorescent protein and blue fluorescent protein were inserted in the wild type strain, and in the constructed mutants respectively by P1 transduction. These were then inoculated in equal proportions in chemostats with appropriate nutrient limiting conditions. The ratio of change of population densities were log transformed and plotted against time. The slope of this line was used as selective coefficients. Relative fitness was measured as (1+ selection coefficient). Each competition experiment was done in duplicate. Error bars represent standard errors to the mean. Students-t test was performed to look for statistically significant differences.

### qPCR analysis for gene glnG and amtB

For qPCR, cells were allowed to grow to stationary phase under appropriate nutrient limiting environments in 10 ml culture volume. 5 ml of the culture was taken for RNA extraction. RNA extraction was done using the RNeasy Mini Kit (Qiagen) as per the manufacturer’s protocol. Extracted RNA was DNase treated using the Turbo DNA-free kit (Ambion) as per the manufacturer’s protocol. The DNA free RNA was run on a 1% gel for visual inspection. 500 ng of RNA (quantified using the Qubit RNA BR assay kit) was used for cDNA preparation using the High Capacity Reverse Transcription Kit (Applied Biosystems). RT-qPCRs were performed using the PerfecTa Sybr Green SuperMix (Quanta Biosciences). The house keeping genes used as reference in the analysis were *cysG* and *hcaT*. The transcript abundance for *glnG* and *amtB* was normalized to the geometrical mean of the levels of *cysG* and *hcaT*. Three biological replicates and 3 technical replicates were used in each case. The averages mentioned are based on biological replicates. Error bars represent standard deviation. Students-t test was performed to look for statistically significant differences.

### Hexadecane assay to measure surface hydrophobicity

A hexadecane assay was used to measure the surface hydrophobicity of the ancestral strain and mutants. 5.0 ml of overnight cultures, grown in low magnesium glucose minimal media, were washed twice with Phosphate Buffer Saline (PBS) solution and were then re-suspended in 1.0 ml of PBS solution. 100 ul of cells was then diluted in 900 ul of PBS and the optical density was measured. This value was noted as C_o_. 200 ul of hexadecane was added to the remainder 900 ul of cells and vortexed for 2 min. This mixture was kept undisturbed at room temperature for 1 h. Two layers are formed, the upper organic layer and lower aqueous layer. Based on the cell surface hydrophobicity, cells get distributed between these two layers. From the lower aqueous layer, 100 ul of cells is taken, diluted in 900 ul of PBS and the optical density was measured. This value was noted as C_h_. Surface hydrophobicity was measured as 100*(C_o_-C_h_)/C_h_. Replicates were done over 2 days, each day with two biological replicates and two technical replicates. The absolute value changed over the 2 days, but the relative values between the wild-type and the mutant did not change. Values are thus shown relative to surface hydrophobicity measured for the wild-type strain under magnesium limiting conditions. Students-t test was performed to look for statistically significant differences.

## Results

### Fitness measurements of evolved clones under reciprocal nutrient limiting conditions

**Fig. 1:**
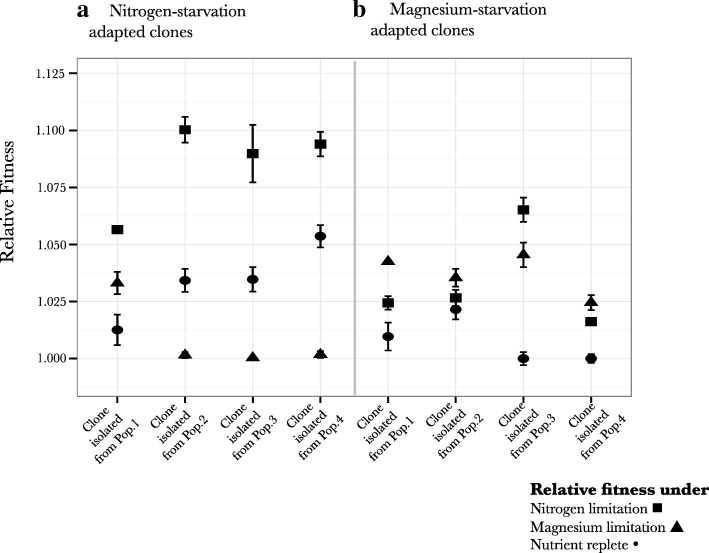
Relative fitness of individual clones on alternate nutrient limiting conditions: A) Relative fitness measurements of nitrogen-limitation adapted clones on limiting nitrogen conditions ■, limiting magnesium conditions ▲, and under nutrient replete conditions • B) Relative fitness measurements of magnesium limitation adapted clones on limiting nitrogen conditions ■, limiting magnesium conditions ▲ and under nutrient replete conditions •.

To investigate the differences between adaptation to nitrogen starvation and magnesium starvation, we first measured relative fitness of evolved clones isolated from the end-point of the experiment on reciprocal nutrient limiting environment i.e. clones that had adapted to limiting nitrogen conditions were tested on limiting magnesium environment and vice-versa (Fig. 1). Three out of four clones evolved under nitrogen limiting conditions did not show any significant increase in relative fitness under limiting magnesium conditions. However, three out of four clones that evolved under limiting magnesium conditions did show substantial increase in relative fitness under nitrogen limiting conditions, which were statistically different from the increase in fitness for these clones under nutrient replete conditions (Fig. 1). This suggests that the adaptive strategies under limiting nitrogen conditions might involve a nutrient specific mechanism, while those under magnesium limitation might involve a more general mechanism.

### Whole-genome population sequencing analysis of populations evolving under nitrogen limiting and magnesium limiting conditions

**Fig. 2:**
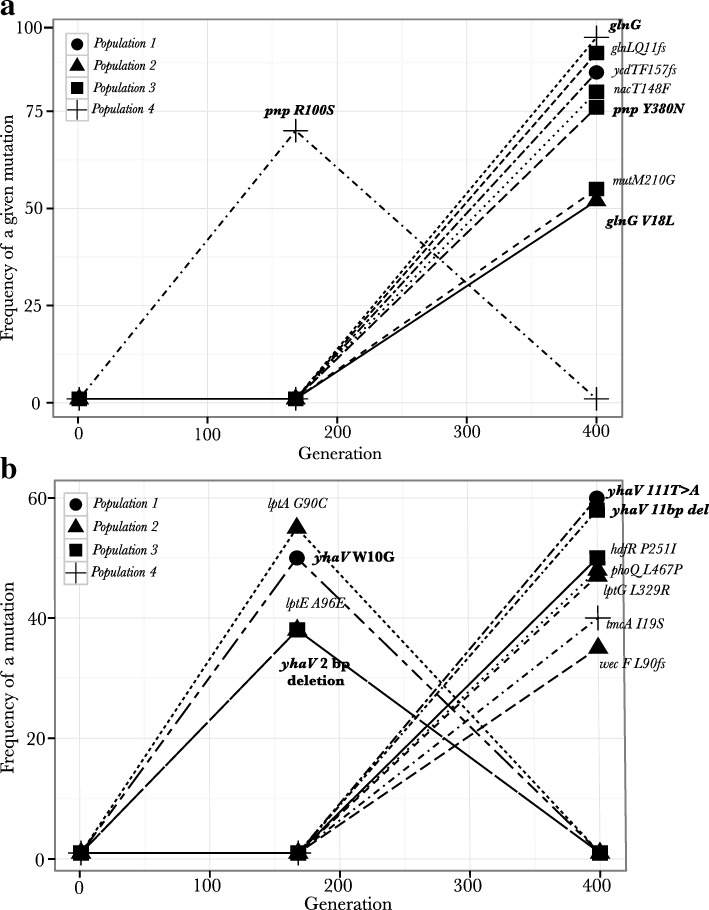
**a**) Potential targets of selection under nitrogen limiting conditions: Trajectories for mutations that reach frequencies of 35% or more for all the populations evolving under nitrogen limiting conditions. Different populations are shown as different shapes. Populations were sequenced at two time points (generations 168 and 400). Genes showing mutations among replicates are written in bold. Different mutations are shown as different types of lines. **b**) Potential targets of selection under magnesium limiting conditions: Trajectories for mutations that reach frequencies of 35% or more for all the populations evolving under magnesium limiting conditions. Different populations are shown as different shapes. Populations were sequenced at two time points (generations 168 and 400). Genes showing mutations among replicates are written in bold. Different mutations are shown as different types of lines

Our previous work identified *glnGL*, that is involved in downstream regulation of operons involved in nitrogen metabolism [32] and plays an important role under nitrogen limiting conditions [33], as an important target of selection under nitrogen limiting conditions with mutations in genes *yhaV*, *lptG* and *phoQ* being important targets of selection under magnesium limiting conditions. We extended the mutational spectra of adaptive mutations under these nutrient-limiting conditions by sequencing the evolved populations at generation 168, and finding other potential targets of selection identified at generation 400. In each case, we identified mutations (SNPs and indels) that reached 35% or higher frequency in each population and compared the dominant mutations at these two time-points (Fig. 2a and b). Below, we only discuss those genes that harbored mutations: i) in at least two replicates under the same nutrient limitation, or ii) were found at both time points in the same population (see Materials and Methods), or iii) reached high frequencies in the population, as they are most likely to be adaptive mutations.

#### Potentially adaptive mutations under nitrogen limitation

##### Genes having mutations among replicate populations:

Besides *glnG*, the other gene that had mutations among replicate populations evolving under nitrogen limiting conditions was *pnp*. The *pnp* gene codes for a polynucleotide phosphorylase protein that plays a role in mRNA and tRNA recycling. Population 3 and population 4 showed non-synonymous mutations in the gene *pnp*. The mutation in population 3 (Y380N) was observed only at generation 400. The mutation seen in population 4 (R100S) was seen at generation 168 reaching a frequency of 70%, but was not seen at generation 400. This population also consisted of clones with mutations in gene *glnG* (V18L) at generation 400, which might have outcompeted the clone with the *pnp* R100S mutation, resulting in this mutation being absent at this time-point. While confirming the mutations observed in gene *glnG* by Sanger sequencing of five clones isolated from the two populations that carried the *glnG* mutation, we also found a mutation 7 bp upstream of this gene (−7G > T) in a single clone from population 2. This mutation was not seen in the population sequencing because of its low frequency.

##### Other potential targets of selection:

At generation 400 the only mutation that reached a high frequency of 85% in population 1 was a single base pair deletion in gene *ycdT*, which codes for a digunaylate cyclase. However population 1 does not show any mutation reaching high frequencies at 168 generations. Population 3 showed a high-frequency mutation in another regulator gene *nac* (80% in the population), which is activated by *glnG* and is itself transcriptional activator of downstream operons for efficient usage of nitrogen. This population also harbored a previously identified 2 bp deletion in *glnL (Q11fs)* that reached a frequency of 90% in the population. Population 3 also showed a mutation in DNA damage repair protein, *mutM*. This resulted in a larger number of mutations reaching higher frequencies in this population as compared to the remaining three populations (see Fig. 2a, 400 generations). A comparison between increase in fitness for populations evolving under nitrogen limitation (Fig. 3 in [28]), and the increase in frequencies of mutations in these populations (Fig. 2a) show a discord at generation 168. Even though we observe all the four populations evolving under nitrogen limitation to have an increase in fitness at this time point, only one population (population 4) shows a mutation that has reached 35% or higher. We think that this discord might be an outcome of the conservative parameters that we have used for detection of mutations in these populations. Re-analyzing the population data with greater coverage is needed to understand the relationship between genotypic diversity and fitness for these populations at these points.

**Fig. 3:**
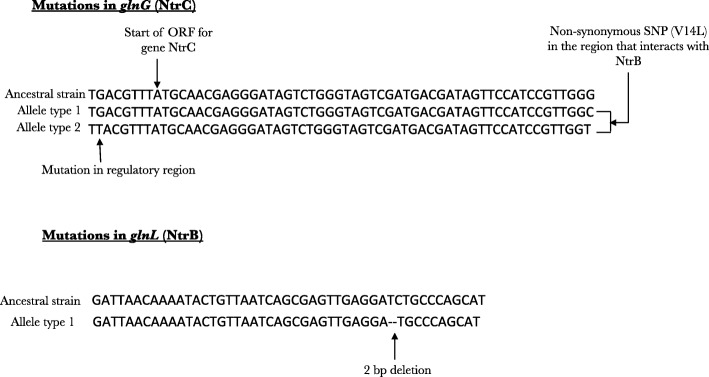
Mutations observed in the dual gene regulator *glnGL* in populations evolving under nitrogen limiting conditions: Population sequencing and local sequencing showed two different *glnG* alleles (*glnG* V18L and *glnG* V18L G-7 T) and a 2 bp deletion in *glnL* to be potential targets of selection under nitrogen limiting conditions

#### Potentially adaptive mutation under magnesium limitation

##### Potential targets of selection:

None of the mutations were found to be repetitive between populations, although as discussed below genes performing similar functions were observed to have mutations in several populations. In population 2 mutations in genes *lptE (A96E)* and *lptA (G90C)* reached frequencies of 38 and 55% respectively, at generation 168. Both of these genes are involved in lipopolysaccharide biosynthesis. A mutation in a different gene from the same operon i.e. *lptG (L329R)* reached a higher frequency at the 400 generations in this population; at which time point both the *lptE* and the *lptA* variants are lost from the population (manually rechecked, Fig. 2b). Population 2 also showed mutations in *phoQ (L467P),* which is a transcriptional regulator that is activated under magnesium limiting conditions and *wecF (L90 fs)* gene, which plays a role in lipid II biosynthesis. In this population, both of these mutations reached frequencies of 48 and 35% respectively. Population 3 showed mutations in *hdfR (P251L)* (repressor of flagellar genes) reaching a frequency of 50% in the population. Population 4 showed no dominant adaptive clone at generation 168. At generation 400 Population 4 showed mutation in *tmcA (I19S)*, which is a cytidine acetyltransferase. This mutation reached 40% in the population.

Overall our analysis showed that regulatory genes that get activated under low nitrogen conditions (*glnG, glnL* and *nac*) and an mRNA-tRNA recycling gene (*pnp*) are important targets of selection under nitrogen limiting conditions, while genes involved in cell-membrane physiology (*lptA, lptE, lptG, wecF, phoQ*) are important targets of selection under magnesium limiting conditions. Under each nutrient limiting regime, among different replicates, we also observed cases of multiple clonal interference events, with one adaptive mutation at a given time point being outcompeted by another adaptive mutation that arose later in the population.

Mutations in gene *yhaV*, previously only identified under magnesium limiting condition*,* on further analysis were also observed in populations evolving under nitrogen limiting conditions (See Material and Methods). breseq analysis showed that many of these were mediated by insertion sequences (Table 1). Under both forms of limitation, complex temporal dynamics were observed for mutations in *yhaV* (Fig. 2b and Table 1). Population 1 evolving under magnesium limitation showed a mutation in gene *yhaV* (W10G) reaching a frequency of 50% in the population at generation 168, however at generation 400 a different mutation in this gene (synonymous mutation at 111 T > A) reached a frequency of 60%, while *yhaV* (W10G) was no longer present in the population. This suggests that different clones having different *yhaV* variant alleles arose in the population, with the latter clones being more fit than the earlier clones. Population 3 showed mutations in *yhaV*, a 2 bp deletion, at generation 168 reaching a frequency of 38% and also a IS5 insertion in *yhaV* at a frequency of 33%. We observed a bigger deletion (11 bp) in the same region at generation 400 reaching a frequency of 58%. Both these deletions appear early in the gene resulting in a truncated protein of 38 amino acids, and thus most likely result in loss of function. Population 3 evolving under nitrogen limiting conditions had IS1 insertion in *yhaV* fixed in the population at both time points. Population 4 evolving under nitrogen limiting conditions showed similar clonal interference patterns of mutations in *yhaV* as was seen in the magnesium limited cultures. IS5 insertion into *yhaV* was observed at generation 168 with this mutant reaching a frequency of 81% in the population; while the same population had an IS2 insertion in *yhaV* reaching 88% in the population at generation 400 (Table 1). Since the same gene had likely loss of function mutations under both these nutrient limiting condition, it is either adaptive under both of these two forms of stress conditions or to the chemostat environment itself. This gene codes for the toxin in the toxin-antitoxin system in *E. coli* and is involved in causing bacteriostasis under unfavorable conditions [34].

**Table 1:** Transposon movements observed during the course of the evolution experiment

Population	Generation	Type of IS element	Overlapping annotation	Frequency
Nitrogen limitation-population 3	168	IS1	*yhaV*	100%
Nitrogen limitation-population 3	400	IS1	*yhaV*	100%
Nitrogen limitation-population 4	168	IS5	*yhaV*	81%
Nitrogen limitation-population 4	400	IS2	*yhaV*	88%
Magnesium limitation-population 3	168	IS5	*yhaV*	33%

### Fitness effects of individual mutations under nutrient-limiting conditions

Strains with individual mutations were constructed to assess the fitness effects of several different putative adaptive mutations. We constructed mutants that individually harbored the two *glnG* alleles that were identified from previous work [28] and in this study (*glnG* V18L and *glnG* V18L G-7 T, Fig. 3) and measured their relative fitness under nitrogen limiting conditions. To assess the fitness effects of adaptive mutations under magnesium limiting conditions, we extended our work to the double mutant *phoQ L467P ΔyhaV,* which showed an increased fitness under magnesium limiting conditions as compared to the ancestral strain [28]. We then examined the *phoQ L467P* and the *yhaV* mutations individually to investigate the fitness effects of these mutations under magnesium limiting conditions.

#### Fitness effect of different *glnG* alleles under nitrogen limiting conditions

**Fig. 4:**
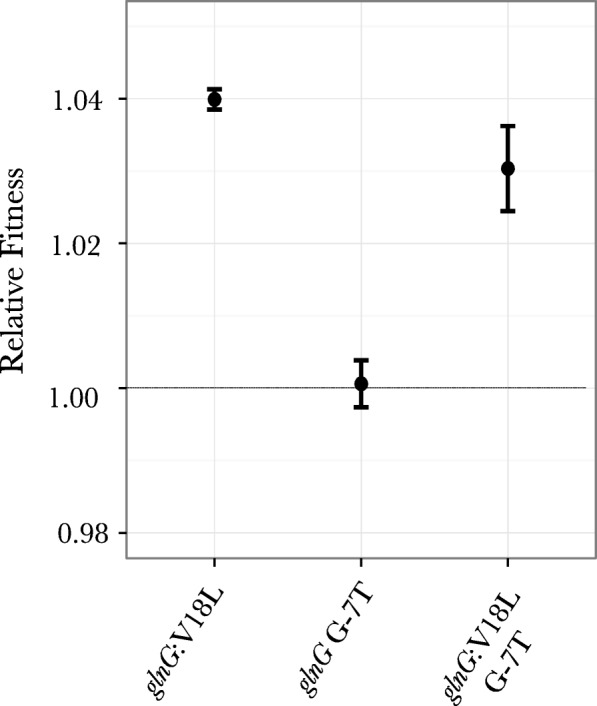
Relative fitness of mutations in *glnG* under nitrogen limiting conditions: Relative fitness of both *glnG* alleles (*glnG* V18L and *glnG* V18L G-7 T) under nitrogen limiting conditions was measured by competing each mutant against the wild-type. Relative fitness measurements of *glnG* G-7 T is also measured and is shown to be neutral under nitrogen limitation. Each experiment was done in a duplicate. Dotted line shows a relative fitness of 1, indicating neutral effect on fitness

Our population genomic analysis and local sequencing of the target gene, done in this study and our previous experiments [28], showed that two different *glnG* alleles (*glnG* V18L and *glnG* V18L G-7 T) and a 2 bp deletion in *glnL (Q11fs)* were selected in populations evolving under nitrogen limiting conditions (Fig. 3). Competition experiments under nitrogen limiting conditions in chemostats showed that the strain harboring mutation *glnG* V18L had a relative fitness of 1.04 ± 0.001 and the strain harboring the second *glnG* allele (*glnG* V18L G-7 T) had a relative fitness of 1.03 ± 0.005 with respect to the ancestor (Fig. 4). To examine the effect of individual SNPs of the second *glnG* allele, we constructed a strain that only had the *glnG* G-7 T mutation. Our results showed that this mutation had no effect on the fitness of the strain under nitrogen limiting conditions, however when in combination with the *glnG* V18L, as is pointed out above (*glnG* second allele), it resulted in a decrease of fitness under nitrogen limiting conditions (*p* = 0.0001) (Fig. 4).

#### Fitness effect of *phoQ* L467P under magnesium limiting conditions

**Fig. 5:**
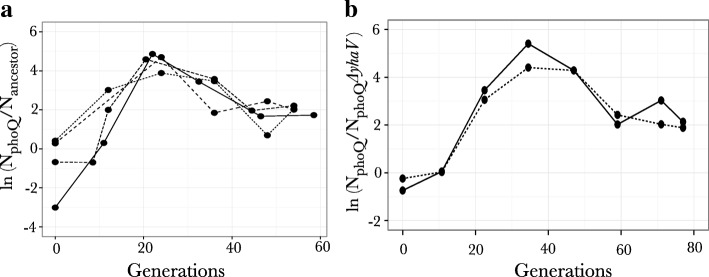
Change in frequency of *phoQ* L467P mutant in competition with **a** wild type strain and **b** *phoQ* L467P*ΔyhaV* double mutant under magnesium limiting conditions: Natural logs of the ratio between the population size of the competing strains is shown. Each experiment was performed as a duplicate and shows in both cases stabilization of the *phoQ* L467P mutant with the **a** wild type strain, even when the starting ratio of the strains are different (1:2 and 1:20) and **b** *phoQ* L467P *ΔyhaV* double mutant in a ratio of ~ 7:1 i.e. ln(2)

A mutation in gene *phoQ* was only observed in one population evolving under limiting magnesium conditions where it reached high frequency (*phoQ L467P)*. Given that the gene *phoQ* is a transcriptional regulator induced under limiting magnesium conditions, we measured the relative fitness of a strain harboring this particular mutation. Our results show that although there appears to be a fitness advantage for this particular mutation, the mutation by itself is not sufficient to eliminate the wild-type strain (Fig. 5a). When competed with the wild-type strain, we initially see an increase and then a subsequent decrease in the frequency of this mutation. Replicate experiments for this particular competition demonstrate that the mutant *phoQ (L467P)* and wild-type strain stabilize at a ratio of ~ 7:1 in the population. The same result is obtained when the starting ratio of the mutant and the wild-type strain is changed from 1:1 to 1:2 and to 1:20 (Fig. 5a), with the ratio between the strains again stabilizing at ~ 7:1 in each case. This phenomenon might either be an outcome of physiological trade-off due to adaptation to magnesium starvation resulting in reduced efficiency of utilization of other resources, or might represent some form of negative frequency-dependent selection.

#### Fitness effect of loss of function *yhaV* mutation with magnesium specific adaptive mutations

**Fig. 6:**
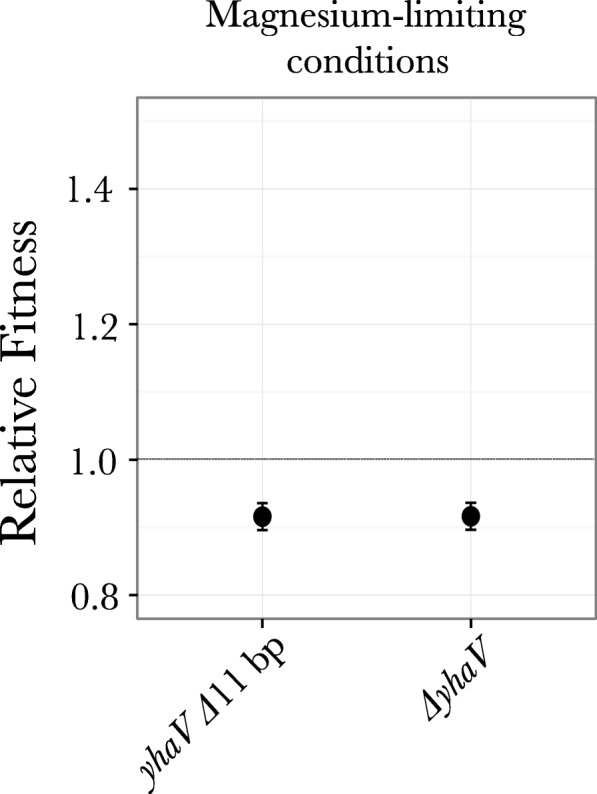
Relative fitness of mutants with *yhaV Δ*11 bp and *ΔyhaV* mutations with respect to the ancestral strain under magnesium-limiting conditions. Relative fitness of mutants was measured by competing each mutant against the wild-type strain under magnesium-limiting conditions. Each experiment was done in a duplicate. Dotted line shows a relative fitness of 1, indicating neutral effect on fitness

The loss of function mutation in gene *yhaV* was observed under both nitrogen limiting conditions and magnesium limiting conditions, indicating that this loss of function mutation is adaptive either as a general stress-response or to chemostat growth. To investigate this, we constructed two mutants: a mutant with the 11 bp out of frame deletion in gene *yhaV* (observed in our evolution experiment) and a mutant with a complete knockout of the gene *yhaV.* Both mutants showed an 8% fitness cost under magnesium limiting conditions (Fig. 6), suggesting that this knockout mutation confers a fitness increase only in the presence of other mutations. This suggestion was corroborated from our previous experiments where the *phoQ* (L467P) *ΔyhaV* double mutant outcompeted the ancestral strain under magnesium-limiting conditions [28]. Interestingly, this competition result showed that when competed against the wild-type ancestral strain, the presence of a deletion of the gene *yhaV* in a strain harboring the *phoQ* L467P resulted in loss of the stabilization phenomenon that was observed when the single mutant *phoQ* L467P was competed against the wild-type (Fig. 5a). To extend on this result and to investigate the nature of this potential sign epistasis we also competed the *phoQ* (L467P) *ΔyhaV* double mutant against the *phoQ* (L467P) single mutant (Fig. 5b) under limiting magnesium conditions. However, in this particular competition experiment we still see stabilization of both the strains at the same ratio as was seen when *phoQ* (L467P) was competed against the wild-type strain (Fig. 5b). Although our results suggests that a loss of function of this particular gene might contribute in reducing potential physiological trade-offs seen in mutations allowing adaptation to magnesium starvation, clearly more work is needed to disentangle these effects and to mechanistically determine why *ΔyhaV* mutants were selected under both magnesium- and nitrogen-limiting conditions*.*

### Fitness trade-offs of adaptive mutations selected under nutrient limiting conditions

#### Fitness trade-offs for adaptation to nitrogen starvation

**Fig. 7:**
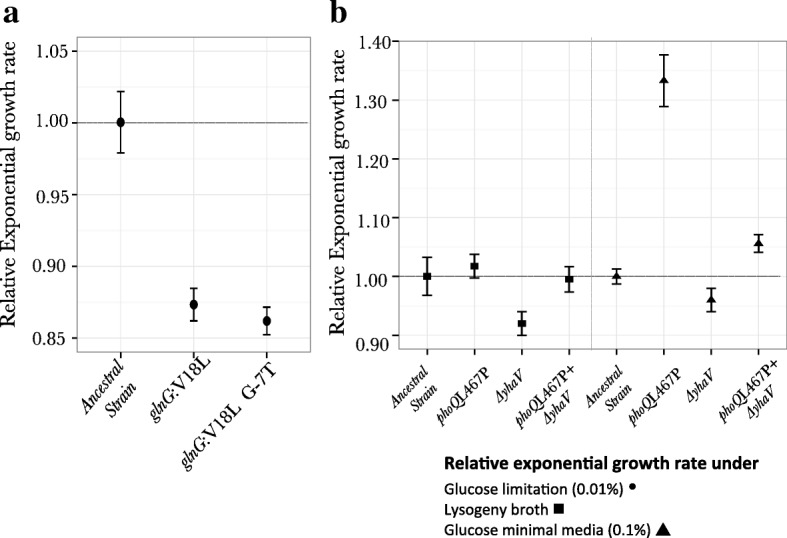
Fitness trade-off for beneficial mutations in different environments: **a** Relative exponential growth rate, with respect to the ancestor (marked with horizontal lines), for clones adapted to limiting nitrogen conditions on glucose limitation (0.01%). **b** Relative exponential growth rate, with respect to the ancestor, for clones adapted to limiting magnesium conditions on nutrient rich media of Lysogeny broth ■ and glucose (0.1%) minimal media ▲

Our results showed that mutations in both the genes *glnG* and *glnL* were selected under nitrogen limiting conditions. Both of these genes are involved in maintaining the C:N ratio in the cell. Thus, to test if the mutations conferring fitness increase under nitrogen limiting conditions have fitness trade-offs under carbon starvation, we measured exponential growth rates (calculated from growth curves) of the *glnG* mutants under glucose-limiting conditions (Fig. 7a). Our results show a significant reduction in exponential growth rate (~ 14%) for both the alleles of *glnG*. This reduction might either be because of these mutations resulting in constitutive expression of the gene, or because of physiological trade-off between adaptation to nitrogen limitation and carbon limitation. We performed gene expression analysis (see below) to differentiate between these two hypotheses.

#### Fitness trade-offs for adaptation to magnesium starvation

Given that the mutants selected under magnesium starvation appear to have altered membranes, and given that the *phoQ* (L467P) mutant resulted in complicated population dynamics, we investigated if this adaptation results in fitness trade-offs under conditions where ample nutrients are present i.e. under nutrient replete conditions. We measured exponential growth rates for wild-type strain, *phoQ* (L467P) mutant, * ΔyhaV* mutant and the *phoQ* (L467P) *ΔyhaV* double mutant in nutrient rich Lysogeny broth (LB) media and in 0.1% glucose minimal media (Fig. 7b). In the nutrient rich LB, no difference was seen in the exponential growth rate of the *phoQ* L467P mutant as compared to the wild-type strain, while an 8% decrease in exponential growth rate was measured for the *ΔyhaV* mutant (Relative exponential growth rate _*phoQ* L467P_ ~ 1.01 ± 0.02; Relative exponential growth rate _*ΔyhaV*_ ~ 0.92 ± 0.02). However in the *phoQ* L467P *ΔyhaV* double mutant, this reduction in growth rate is no longer seen (relative exponential growth rate _*phoQ* L467P *ΔyhaV*_ ~ 0.99 ± 0.02). Thus, even if no fitness trade-off was seen for the *phoQ* L467P mutant or the *phoQ* L467P *ΔyhaV* double mutant, we clearly see epistasis at the level of fitness between these two mutations in nutrient rich media. Interestingly, we observe an increase in the exponential growth rate of the *phoQ* L467P mutant in the 0.1% glucose minimal media (relative exponential growth rate ~ 1.33 ± 0.04) and a small yet significant decrease in the exponential growth rate of the *ΔyhaV* mutant (Relative exponential growth rate ~ 0.96 ± 0.02). The relative exponential growth rate for the *phoQ* L467P *ΔyhaV* double mutant in this media was measured to be 1.05 ± 0.01. Again, like in the nutrient rich LB we see fitness epistasis between these two mutations as in 0.1% glucose minimal media.

### Adaptive strategy under nitrogen limitation involves increased expression of *glnG* and *amtB* genes

**Fig. 8:**
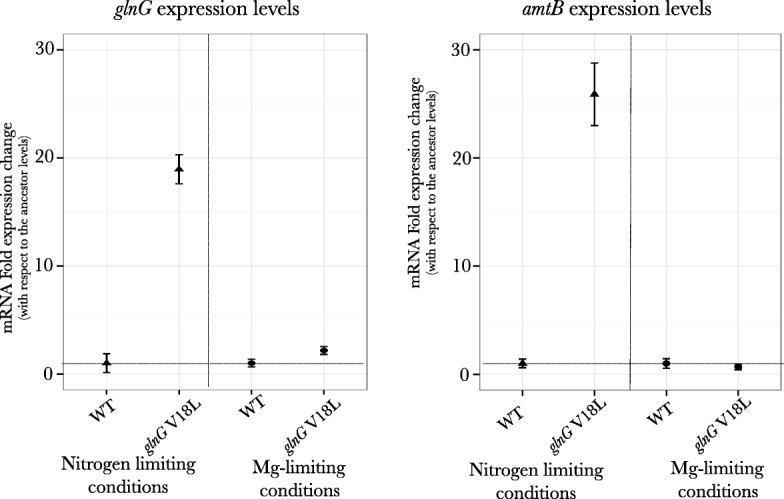
qPCR analysis for genes *glnG* and *amtB* in mutant adapted to nitrogen limitation: Gene expression analysis for genes *glnG* and *amtB* for wild-type ancestral strain and mutant with *glnG* V18L mutation under nutrient limiting conditions of nitrogen limitation and magnesium limitation. Increased expression levels for both the genes were seen only under limiting nitrogen conditions. Relative expression levels with respect to wild-type (horizontal line) plotted

To understand the mechanistic basis of the mutations selected under nitrogen limitation, qPCR analysis was performed for genes *glnG* and *amtB* in the *glnG* V18L mutant. The analysis was also done under magnesium limiting conditions as a control. Expression levels of *glnG* is primarily regulated by the *glnL* coded NtrB protein. This gene carried a mutation that reached fixation in one of replicates evolving under nitrogen limiting conditions. The *amtB* gene codes for the protein that functions as the ammonium ion transporter and is regulated by *glnG*. Our qPCR analysis showed that in comparison to the wild-type the *glnG* V18L mutant had a 18-fold higher expression of the *glnG* gene under nitrogen limiting conditions, resulting in a 27 fold increase in the expression of the *amtB* gene (Fig. 8). If the increase in expression of these genes is constitutive in nature (loss of regulation), it is expected that this increase will still be observed in other environments as well. However, no difference in expression levels for these two genes was seen between the wild-type strain and the mutant under magnesium limiting conditions, suggesting that the increased expression is still regulated and has not become constitutive.

### Adaptive strategies to magnesium limitation involves change in lipopolysaccharide physiology

**Fig. 9:**
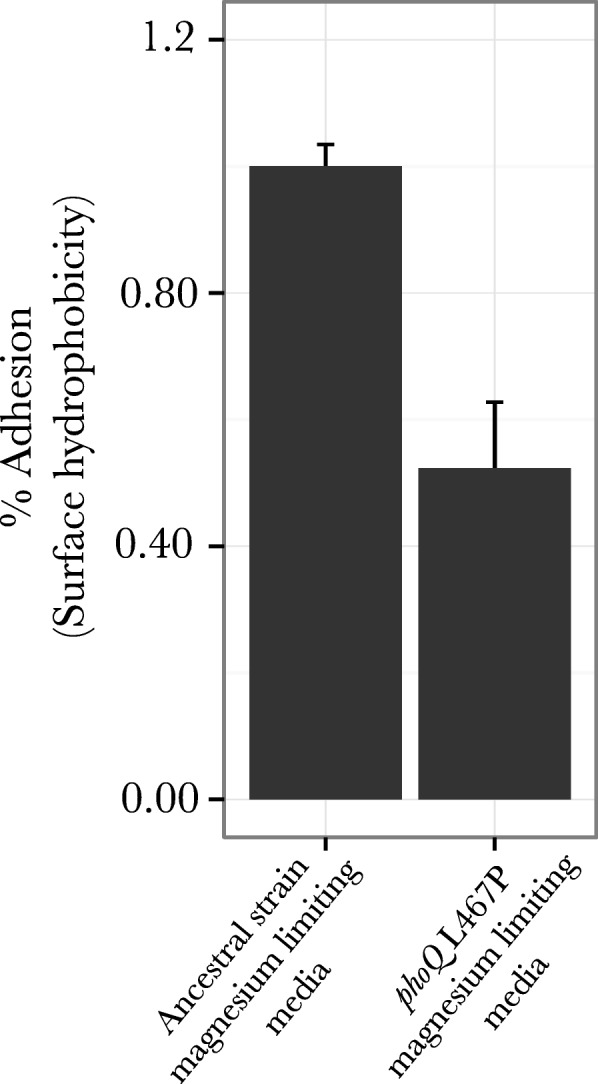
Surface hydrophobicity analysis for mutants adapted to limiting magnesium conditions: Hexadecane assay was performed to measure surface hydrophobicity for wild-type ancestral strain and *phoQ* L467P mutant under magnesium limiting conditions

Given that both the population genomic analysis and strain construction results showed that proteins involved in lipopolysaccharide synthesis (*lptE, lptA, lptG, phoQ, wecF* and *phoQ* (L467P)) are important targets of selection under magnesium starvation, we performed cell membrane surface hydrophobicity assays using hexadecane to understand the nature of this adaptation. Under magnesium starvation, the lipopolysaccharide component (LPS) of the outer-membrane becomes a primary reservoir of magnesium ions. Under these conditions, LPS is produced using alternate positively charged and polar sugar moieties that result in lower magnesium ion requirement for LPS stabilization [35]. Consequently, the increase in the number of these positively charged polar sugar molecules in LPS results in lowering of the surface hydrophobicity. Smit et al. [36] showed similar results where the surface hydrophobicity of yeast cells was reduced under limiting magnesium conditions. Consistent with these physiological expectations, our results show that under magnesium limiting conditions the *phoQ* L467P mutant has a lower surface hydrophobicity as compared to the wild-type (Fig. 9, *p* = 0.001). This observation suggests that there is a larger proportion of polar sugar residues in the LPS molecule of the mutant as compared to the wild-type strain, under magnesium limiting conditions. Consequently, there is an even lower need for magnesium ions to stabilize the LPS. Three of the other genes mentioned as potential targets of selection (*lptE*, *lptA* and *lptG*) are all involved in LPS assembly. These results are highly suggestive that mutations being selected under magnesium limitation might result in change in LPS physiology, such that lower amounts of magnesium are needed for stabilization of LPS allowing magnesium to be used in alternate cellular functions.

## Discussion

### Clonal interference events dominate evolutionary trajectories under nutrient-limiting conditions

The use of the population sequencing approach over two time points during the course of our evolution experiment allowed us the investigate, at a coarse level, the clonal interference events taking place in the populations evolving under nutrient limiting conditions. Under nitrogen-limiting conditions, the mutation in gene *pnp* (R100S) observed at generation 168 was replaced by the *glnG* (V18L) mutation at generation 400. Adaptive mutations in the genes in the *lpt* operon also showed similar successive replacements events. Specifically, the *lptG (L329R)* mutant outcompeted the *lptE (A96E)* and *lptA (G90C)* in the same population under magnesium limiting conditions. Overall these findings suggest that even under conditions of single nutrient-limitation in chemostats, where the selective pressure and population-size is expected to remain constant throughout the experiment, evolutionary trajectories might be very complicated and involve multiple clonal interference events. Our results further demonstrate that these events can be an outcome of beneficial mutations in genes with different physiological functions (as seen under nitrogen limitation) or through mutations in genes with similar functions (as seen under magnesium limitation). Not only does this result add to previous observations of clonal interference events under limitation of macronutrients [11, 37, 38], it demonstrates that clonal interference is also prevalent under limitation of major cations.

### Adaptation to nitrogen-limitation occurs by nutrient-specific changes, while adaptation to magnesium-limitation occurs by more general changes

Our results suggest two different patterns of evolutionary response under the two nutrient-limiting conditions. Clones adapted to nitrogen limitation showed nutrient-specific evolutionary responses. Thus, for this set of clones the increase in relative fitness was only observed under nitrogen limitation but not under magnesium limitation. In contrast, adaptation to magnesium limitation also resulted in an increase in relative fitness under limiting nitrogen conditions suggesting that this adaptation might proceed via a more general stress response route. How can these differences be accounted for? Under nitrogen limiting conditions, the mutations identified are expected to result in an increase in uptake rate of the limiting nutrient by increased production of transporter proteins. Similar results have been observed for carbon limitation [15–17, 39] as well as for phosphorous limitation [20]. In contrast, under magnesium limitation besides the mutation in regulator gene *phoQ*, an alternate form of adaptive strategy is observed where mutants with changes in genes involved in cell membrane biogenesis and flagellar operon are selected. These mutations are expected to have pleiotropic effects and more likely to increase relative fitness in other nutrient limiting environments. In this case, this potentially involved the redistribution of magnesium from the LPS to other molecules.

### Genetic changes in nutrient specific regulation under nitrogen limiting conditions

The NtrB protein, coded by gene *glnL,* responds to extracellular nitrogen levels and modulates activity of NtrC (encoded by *glnG),* through phosphorylation-dephosphorylation steps. NtrC induces the expression of downstream operons that are mainly involved in scavenging of nitrogen sources from the environment and in regulating amino acid synthesis. Under nitrogen limiting conditions, mutations in both these genes were selected. The non-synonymous SNP in the gene *glnG* coding for protein NtrC was observed in the region of the protein that interacts with protein NtrB (*glnL*), while the 2 bp deletion in the gene coding for NtrB protein results in a premature stop codon. Our mutant reconstruction result showed that both *glnG* alleles increase relative fitness under nitrogen limiting conditions. Furthermore, gene expression analysis showed that this increase was due to increased expression levels of the *glnG* gene and the *glnG* controlled gene *amtB* that encodes an ammonium ion transport protein. Thus, it is likely that the adaptive response mainly involves increased uptake of the limiting nutrient; although, it is also possible that increased *glnG* expression level might result in more efficient usage of cellular nitrogen, making the cell survive better under nitrogen limiting conditions.

It is interesting to contrast our result with other studies that have looked at adaptive responses to nitrogen limitation. Hong et al. [19] showed that adaptive responses to nitrogen limitation in yeast results in copy number variation for the transporter protein for the particular nitrogen source. Jezequel et al. [18] showed that one of the adaptive responses to nitrogen limitation for prokaryote *Acinetobacter baylyi* involved a mutation in the gene *glnK.* The protein encoded by this gene interacts with NtrB to induce expression of NtrC, the latter two being targets of selection in our experiments.

### Change in cell-membrane physiology as an adaptive strategy under magnesium limiting conditions

Across the four replicates evolving under magnesium limitation, we found several genes involved in cell-membrane physiology to be targets of selection. This result is expected because magnesium ions play an important role in stabilization of the cellular membrane. As early as 1969, Fiil and Branton [40] showed that cells growing in magnesium deficient environment change their cell membrane structure. Importantly these authors also showed that these cells had the same the amount of magnesium ion present per cell as compared to cells grown in magnesium replete conditions. This suggests that the cell-membrane structures, especially the LPS, are important sources of magnesium ions under magnesium starvation [41]. Several studies have shown that under magnesium limiting conditions, an immediate physiological change seen in the cell is the addition of polar positively charged sugar moieties to the LPS, releasing the magnesium attached to these molecules. These magnesium ions can potentially be used for alternate processes. Our observation that genes regulating cell-membrane physiology, specifically the LPS, are important targets of selection further strengthens this concept. The surface hydrophobicity measurements for the *phoQ* (L467P) was much lower compared to the wild-type strain. This finding suggests that in this mutant, even less magnesium ions are needed for LPS stabilization, allowing a greater part of this reservoir to be used for alternate cellular processes.

## Conclusions

In conclusion, our study has identified two different mechanisms for regulation of elemental economization, increased uptake and redistribution of the limiting nutrient. Major targets of selection under nitrogen limitation were genes encoding regulatory proteins that control expression levels of genes involved in nitrogen-scavenging. The observed mutations caused increased expression of nitrogen transporter proteins, most likely resulting in increased uptake of nitrogen. In contrast, major targets of selection under magnesium limitation were genes regulating outer-membrane lipopolysaccharide synthesis. Since the lipopolysaccharide acts as a reservoir for magnesium ions under magnesium limiting conditions, it is plausible that the adaptive strategy under magnesium limitation involves redistribution of magnesium from the cell wall to other processes.

We also discovered another gene, *yhaV,* where knock-out mutations are commonly selected in chemostat cultures irrespective of the which nutrient is limiting. Mutations in this gene and in *rpoS* seem to be selected in chemostat environments where slow growth is maintained by nutrient limitation.

## Additional file

Additional file 1: **Table S1.** Accession numbers for whole-genome fastq files for the different populations sequenced. (DOCX 14 kb)
