# Supplementary material for: Different adaptive strategies in E. coli populations evolving under macronutrient limitation and metal ion limitation
Source: BMC Evol Biol. 2018 May 18;18:72. doi: 10.1186/s12862-018-1191-4 (PMC5960147; doi:10.1186/s12862-018-1191-4)
Supplement: Supplementary file 1 — Table S1. Accession numbers for whole-genome fastq files for the different populations sequenced. (DOCX 14 kb) [file 12862_2018_1191_MOESM1_ESM.docx]

Table S1: Accession numbers for whole-genome fastq files for the different populations sequenced

| **Population sequenced** | **Time Point (Generation)** | **Accession number** |
| --- | --- | --- |
| Nitrogen-limited population 1 | 168 | SAMN07184674 |
| Nitrogen-limited population 1 | 400 | SAMN07184675 |
| Nitrogen-limited population 2 | 168 | SAMN07184676 |
| Nitrogen-limited population 2 | 400 | SAMN07184677 |
| Nitrogen-limited population 3 | 168 | SAMN07184678 |
| Nitrogen-limited population 3 | 400 | SAMN07184679 |
| Nitrogen-limited population 4 | 168 | SAMN07184680 |
| Nitrogen-limited population 4 | 400 | SAMN07184681 |
| Magnesium-limited population 1 | 168 | SAMN07184682 |
| Magnesium-limited population 1 | 400 | SAMN07184683 |
| Magnesium-limited population 2 | 168 | SAMN07184684 |
| Magnesium-limited population 2 | 400 | SAMN07184685 |
| Magnesium-limited population 3 | 168 | SAMN07184686 |
| Magnesium-limited population 3 | 400 | SAMN07184687 |
| Magnesium-limited population 4 | 168 | SAMN07184688 |
| Magnesium-limited population 4 | 400 | SAMN07184689 |
